# Identification a novel *MYOC* gene mutation in a Chinese family with juvenile-onset open angle glaucoma

**Published:** 2010-08-25

**Authors:** Xin Zhao, Chaoshan Yang, Yi Tong, Xiaohui Zhang, Liang Xu, Yang Li

**Affiliations:** 1 Beijing Institute of Ophthalmology, Beijing Tongren Eye Center, Beijing Tongren Hospital, Capital Medical University, Beijing Ophthalmology & Visual Sciences Key Laboratory, Beijing, China; 2 LuShan Hospital, Henan, China; 3 The First Affiliated Hospital, Fujian Medical University, Fuzhou, Fujian, China

## Abstract

**Purpose:**

To describe the clinical and genetic findings in one Chinese family with juvenile-onset open angle glaucoma (JOAG).

**Methods:**

One family was examined clinically and a follow-up took place 5 years later. After informed consent was obtained, genomic DNA was extracted from the venous blood of all participants. Linkage analysis was performed with three microsatellite markers around the *MYOC* gene (D1S196, D1S2815, and D1S218) in the family. Mutation screening of all coding exons of *MYOC* was performed by direct sequencing of PCR-amplified DNA fragments and restriction fragment length polymorphism (RFLP) analysis. Bioinformatics analysis by the Garnier-Osguthorpe-Robson (GOR) method predicted the effects of variants detected on secondary structures of the MYOC protein.

**Results:**

Clinical examination and pedigree analysis revealed a three- generation family with seven members diagnosed with JOAG, three with ocular hypertension, and five normal individuals. Through genotyping, the pedigree showed a linkage to the *MYOC* on chromosome 1q24–25. Mutation screening of *MYOC* in this family revealed an A→T transition at position 1348 (p. N450Y) of the cDNA sequence. This missense mutation co-segregated with the disease phenotype of the family, but was not found in 100 normal controls. Secondary structure prediction of the p.N450Y by the GOR method revealed the replacement of a coil with a β sheet at the amino acid 447.

**Conclusions:**

Early onset JOAG, with incomplete penetrance, is consistent with a novel mutation in *MYOC*. The finding provides pre-symptomatic molecular diagnosis for the members of this family and is useful for further genetic consultation.

## Introduction

Primary open-angle glaucoma (POAG;OMIM 137760) is one of the leading causes of blindness in the world [1]. It is a neurodegenerative disorder characterized by progressive excavation of the optic discs due to loss of retinal ganglion cells. It is usually associated with elevation of intraocular pressure (IOP) [2]. Based upon the age of diagnosis, primary open-angle glaucoma can be sub-classified to either juvenile-onset primary open-angle glaucoma (JOAG) or adult-onset primary open-angle glaucoma. JOAG is a relatively rare form of primary open angle glaucoma that occurs in children and young adults. The exact age boundary for juvenile-onset varies from one study to the next, but it usually falls between 35 and 40 years of age [2].

Strong evidence indicates that genetic factors play a role in the pathogenesis of glaucoma. About 30%–56% of patients with glaucoma or ocular hypertension (OHT) have a positive family history; first-degree relatives of POAG patients are seven to ten times more likely to have POAG, compared with the general population [3,4]. Genetically, most POAG cases follow a complex (non-Mendelian) pattern of inheritance, which manifests clinically in adulthood (>40 years). However, juvenile-onset open-angle glaucoma typically shows an autosomal dominant inheritance [2-4]. To date, three genes, namely myocilin (*MYOC*), optineurin (*OPTN*), and WD repeat-containing protein 36 (*WDR36*), have been reportedly linked to POAG [5-10]. *MYOC* (OMIM 601652) was the first gene to be identified as responsible for POAG. Mutations in *MYOC* account for over 8% of JOAG and 3%–4% of adult-onset POAG [11,12].

*MYOC,* consisting of three exons, encodes 504 amino acid residues. Myocilin is an acidic protein that contains an NH_2_-terminal myosin-like domain and a COOH-terminal olfactomedin-like domain [6]. Almost 80 mutations have been found in *MYOC* and about 90% of the mutations are located in the olfactomedin-like domain encoded by exon3 [6,11-30].

In this study, we describe the clinical findings in a Chinese family with a novel *MYOC* mutation.

## Methods

### Patients and DNA sample collection

This study was performed according to the tenets of the Declaration of Helsinki for research involving human subjects. This study was approved by the Beijing Tongren Hospital Joint Committee on Clinical Investigation. After informed consent was obtained, all participants underwent ophthalmologic examination including bilateral best corrected visual acuity using E decimal charts, slit-lamp biomicroscopy inspection of the anterior chamber, intraocular pressure (IOP) measurement by applanation tonometry (Goldmann), anterior chamber angle evaluation by gonioscopy (Goldmann), and fundus examination with a 66-diopter VOLK lens. Most members were clinically followed for five years, from 2004 to 2009. Some individuals underwent Octopus’s perimeter examination. Diagnosis of POAG was based on the observation of at least two of the following abnormalities: characteristic glaucomatous optic disc changes, characteristic glaucomatous visual field defects, and high intraocular pressure (>21 mmHg) in the presence of a normal open anterior chamber angle. Characteristic glaucomatous optic disc changes include vertical cup-disc (c/d) ratio of 0.7 or more, notching of the neutral rim, and disc hemorrhage. Subjects were sub-classified JOAG if the diagnosis of POAG was made before 35 years of age. Individuals with intraocular pressure greater than 22 mmHg but with no characteristic optic disc damage or visual field impairment were defined as ocular hypertension. Unaffected people had IOP in the normal range (≤21 mmHg) and optic nerves presented normal in appearance.

### Linkage analysis

Genotyping and linkage analysis were performed with three microsatellite markers (D1S196, D1S2185, and D1S218) around the *MYOC* gene in the family. The fine mapping primer sequences were obtained from the GDB Human Genome Database. LOD scores were calculated for the two markers by two-point linkage analysis using linkage package 5.2. We modeled the disease as an autosomal dominant trait with reduced penetrance. Pedigree and haplotype maps were constructed using Cyrillic version 2.0 software.

### Mutation screening of *MYOC*

Peripheral blood was obtained by venipuncture and genomic DNA was extracted according to standard protocols. The entire coding region of *MYOC* was amplified by polymerase chain reaction (PCR) from genomic DNA. Primers for three exons and exon-intron boundaries of *MYOC* were designed by the Primer3 program. These primer sequences are presented in Table 1. For direct sequencing, PCR products were purified (Shenneng Bocai PCR purification kit; Shenneng, Shanghai, China). An automatic fluorescence DNA sequencer (ABI, Prism 373A; Perkin Elmer, Foster City, CA), used according to the manufacturer’s instructions, was used to sequence the purified PCR products in both forward and reverse directions. DNAssist Version 1.0 compared nucleotide sequences with the published DNA sequence of *MYOC* (GenBank NM_000261). For the *MYOC* gene, cDNA numbering +1 corresponded to the A in the ATG translation initiation codon of *MYOC*.

**Table 1:** PCR primers used in this study.

**Primer**	**Forward (5'-3')**	**Reverse (5'-3')**	**Tm (°C)**	**Product size (bp)**
exon1	CTCTGTCTTCCCCCATGAAG	AGCAGGTCACTACGAGCCATA	62	785
exon2	TAGTCAATCCTTGGGCCATT	ACCACGTGGGCACAAAAG	60	561
exon3-1	CTTCCGCATGATCATTGT	CTTCCGCATGATCATTGT	58	352
exon3-2	ATACTGCCTAGGCCACTGGAA	CCGCTATAAGTACAGCAGCATGAT	58	440
exon3-3	GCCTTCATCATCTGTGGCAC	CAGGCAGCTTTGACTGCTTT	58	342

### Restriction fragment length polymorphism (RFLP) analysis

To confirm the variations found in the sequencing, restriction endonuclease HindII (New England Biolabs, Ipswich MA) was used in all available family members and in 100 normal control subjects. The reaction was performed in a 10 μl volume containing 9.4 μl PCR product, 0.1 μl BSA (100 μg/ml), and 0.5 μl enzyme (10 U/μl). After incubating the reaction overnight at 37 °C, the entire digest was run on a 1% agarose gel and visualized under ultraviolet light.

### Bioinformatics analysis

Garnier-Osguthorpe-Robson (GOR) software was used to predict the effect of the mutation on the secondary structure of MYOC [31]. This method infers the secondary structure of a sequence by calculating the probability for each of the four structure classes (helix, sheet, turn, and loop) based on the central residue and its neighbors from the calculated matrices.

## Results

### Clinical findings

**Figure 1:**
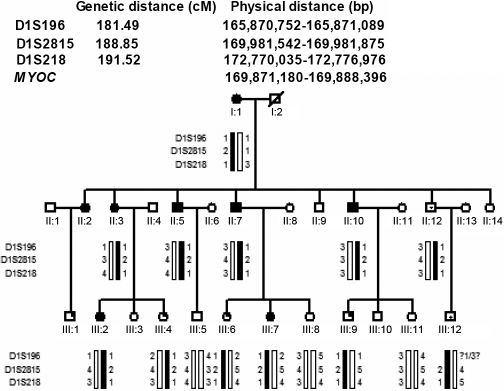
Family structure and haplotype analysis of a Chinese family with JOAG. Pedigree and haplotype analysis of the family with JOAG showed segregation with three microsatellite markers on chromosome 1, listed in descending order from the centromeric end. Squares indicate males; circles indicate females; slashed symbols indicate deceased; solid symbols indicate affected; open symbols indicate unaffected; symbols with upper left filled-in quadrant indicate members with ocular hypertension; symbols with dot in the center indicate carriers.

**Figure 2:**
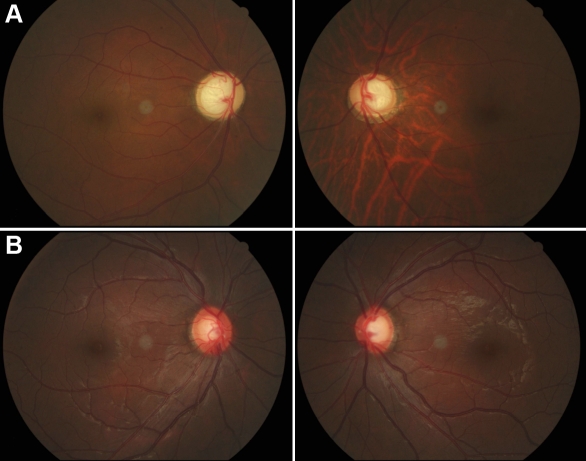
Fundus appearances of patients with JOAG. **A**: Fundus images of II:7 showed late-stage glaucomatous cupping of the optic disc. **B**: Fundus images of III:7, who was confirmed to have glaucoma in 2009, presented early glaucomatous appearances of the optic disc.

We have identified a three- generation family diagnosed with JOAG. The inheritance pattern in this family appeared to be autosomal dominant (Figure 1). After clinical examinations and hospital records reviewing, six individuals of this pedigree were found to have glaucoma in 2004. The patient in the first generation had not received any treatment and totally lost her sight before the age of 35. The remaining five patients underwent trabeculectomies in both eyes. The mean onset age of these patients was 27.42 years (ranging from 20 to 31 years old), which was consistent with juvenile glaucoma. All patients experienced elevated IOP (32–50 mmHg) and most of them presented typical late stage glaucoma changes in the optic disc and in the visual field (Figure 2A). In 2004, six members were diagnosed with ocular hypertension (IOPs were higher than 22 mmHg) but without optic disc or visual field changes. A five-year follow-up was conducted with fifteen of the seventeen individuals and their blood samples were collected for further genetic analysis. At the 5-year follow-up, two ocular hypertension patients (Figure 1; III:2 and III:7) were newly diagnosed with glaucoma due to their elevated IOP, enlarged cup/disc ratio of the optic disc, and early visual field changes in 2009 (Figure 2B) . Detailed clinical information of the pedigree is summarized in Table 2.

**Table 2:** Clinical features of individuals of this pedigree with JOAG.

**Pedigree number**	**Gender/ Age year**	**Onset Age year**	**BCVA OD/OS (2004)**	**BCVA OD/OS (2009)**	**Maxium IOP mmHg**	**IOP (OD/OS) (2004) mmHg**	**IOP (OD/OS) (2009) mmHg**	**Optic Disc (C/D) (OD/OS) (2004)**	**Optic Disc (C/D) (OD/OS) (2009)**	**Medical therapy (OD/OS)**	**Diagnosis (2004)**	**Diagnosis (2009)**	**N450Y**
II:4	F/79	20	NLP	NLP	NA	N/A	NA	1.0/1.0	1.0/1.0	NMT	JOAG	JOAG	Yes
III:1	F/58	30	0.8/0.8	NA	45/50	18/16	NA	0.6/0.6	NA	S/S	JOAG	NA	NA
III:3	F/56	28	0.2/0.2	0.2/0.2	50/60	10/14	14/14	0.9/0.9	0.9/0.9	S/S	JOAG	JOAG	Yes
III:5	M/52	31	0.2/0.2	0.8/0.8	53/40	20/20	16/15	0.8/0.4	0.8/0.4	S/S	JOAG	JOAG	Yes
III:7	M/49	29	0.1/0.1	0.1/0.1	52/56	15/15	22/22	0.9/0.9	0.9/0.9	S/S	JOAG	JOAG	Yes
III:10	M/45	28	0.1/0.1	0.1/0.1	55/55	31/21	35/28	0.9/0.9	0.9/0.9	S/S	JOAG	JOAG	Yes
III:12	M/39		0.8/0.8	0.8/0.8		21/16	21/21	0.2/0.2	0.2/0.2		Normal	Carrier	Yes
IV:1	M/30		1.0/1.0	NA		24/24	NA	0.4/0.4	NA		OHT	NA	NA
IV:2	F/22	22	1.0/1.0	1.0/1.0	30/32	22/22	28/26	0.4/0.4	0.7/0.5	M/M	OHT	JOAG	Yes
IV:3	F/19		1.0/1.0	1.0/1.0		22/22	NA	0.5/0.5	NA		OHT	OHT	Yes
IV:4	M/21		1.0/1.0	1.0/1.0		18/18	16/16	0.4/0.4	0.4/0.4		Normal	Normal	No
IV:5	F/23		1.2/1.2	1.2/1.2		25/25	25/25	0.3/0.3	0.3/0.3		OHT	OHT	Yes
IV:6	F/22	22	1.0/1.0	1.0/1.0	34/32	26/26	34/32	0.5/0.5	0.7/0.7	M/M	OHT	JOAG	Yes
IV:7	F/17		1.0/1.0	1.0/1.0		14/14	18/18	0.2/0.2	0.2/0.2		Normal	Normal	No
IV:9	M/22		1.0/1.0	1.0/1.0		24/20	26/20	0.5/0.5	05/0.5		OHT	OHT	Yes
IV:10	F/16		1.0/1.0	1.0/1.0		15/15	16/17	0.2/0.2	0.2/0.2		Normal	Normal	No
IV:12	M/16		1.2/1.2	1.2/1.2		19/20	17/17	0.2/0.2	0.2/0.2		Normal	Carrier	Yes

Abbreviations: M, male; F, female; BCVA, best-correct visual aucuity; OD, right eye; OS, left eye; NLP, no light peception; IOP, introocular pressure; C/D, cup disc ratio; NMT, no medical therapy; NA, unavailable; S, surgery; M, medical therapy; OHT, ocular hypertension, JOAG, juvenile-onset angle glaucoma.

### Genotyping results

The family was genotyped with three STRP markers located around the *MYOC* gene in the chromosome 1q24–25 region. The marker results for D1S218 and D1S2815 were fully informative for linkage. There was no affected (glaucomatous patients and ocular hypertension patients) recombinant for either of the two makers (Figure 1). Two clinical unaffected individuals (II:12 and III:12), however, were found to be carrying the affected haplotype. Therefore, the disease penetrance appeared incomplete in this pedigree. Two-point LOD scores for D1S2815 and D1S218 with 80% penetrance were 2.40 (θ=0.0) and1.63 (θ=0.0), respectively.

### Mutation analysis

**Figure 3:**
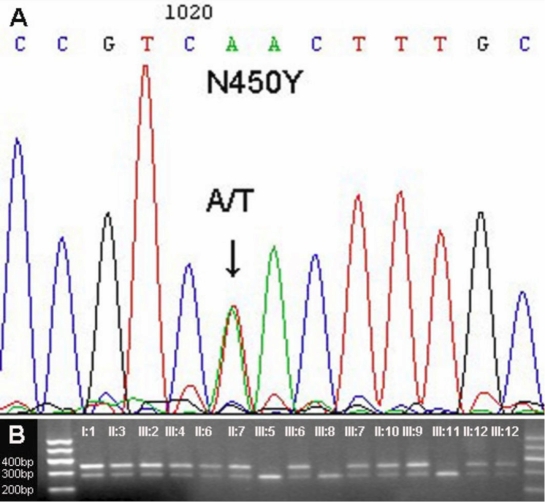
DNA sequence chromatograms and co-segregation analysis of the p.N450Y mutation with disease phenotype. **A**: Heterozygote sequence (sense strand) shows an A/T transition in codon 450 that changed asparagine (AAC) to tyrosine (TAC). **B**: Restriction fragment length analysis shows the p.N450Y mutation abolishing a HindII site co-segregated with JOAG patients, ocular hypertensions, and the carriers (342 and 279 bp), but not with unaffected individuals (279 bp).

By direct sequencing of three exons of *MYOC*, we found a novel base change (A→T) at position 1348 of *MYOC* cDNA, replacing asparagine with tyrosine at amino acid 450 residue (Figure 3A). This heterozygous missense mutation abolished a HindII restriction site that segregated with all affected members and ocular hypertension individuals in this Chinese family, but that was not detected in 100 unrelated normal controls. As observed in the genotyping, two clinical unaffected individuals (II:12 and III:12) carried the mutation as well (Figure 3B).

### Prediction of two-dimensional structure

**Figure 4:**
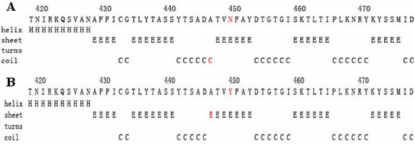
The effect of p. N450Y on the secondary structure of MYOC using the GOR method. **A**: The secondary structure of wild type MYOC around the site N450. **B**: The secondary structure of mutant Y450 of MYOC of the corresponding region.

Using the GOR method, the results for secondary structure prediction suggested that the mutant MYOC450Y replace a coil “C” with a β sheet “E” at amino acid 447 Figure 4).

## Discussion

This study described a Chinese family with clinically diagnosed juvenile-onset open angle glaucoma. By screening the *MYOC* gene, we identified a novel heterozygous missense mutation p. N450Y in the pedigree. The mutation p. N450Y co-segregated with all glaucoma patients and ocular hypertension individuals, but was not detected in 100 normal controls.

*MYOC* was the first disease-causing gene identified for POAG and almost 80 mutations have been reported [6,11-30]. Mutations in *MYOC* are racial/ethnic specific and some of them have been found only in a specific region [6,11-30]. So far, 11 *MYOC* mutations have been identified in Chinese patients or pedigrees and seven of them were Chinese specific (Table 3) [19,20,22,23,25,26,28].

**Table 3:** Mutations in *MYOC* identified in Chinese families or patients.

**Mutation**	**Location**	**Case control**	**Family-base**	**Phenotype**	**Proband age at diagnosis**	**Country/ethnicity**	**Reference**
R91X	Exon1	Yes		NA		China	[19]
C245Y	Exon3	Yes	Yes	JOAG	16	China	[22,23]
G252R	Exon3	Yes	Yes	JOAG	29	Caucasian, China	[12,20]
E300K	Exon3	Yes		NA	NA	China	[19,22]
S313F	Exon3	Yes		NA	NA	China	[22]
Q337X	Exon3		Yes	JOAG	40**	China	[28]
S341P	Exon3		Yes	JOAG	24	China, Korean	[14,26]
T353I*	Exon3	Yes		NA	NA	Asian	[19,22]
P370L	Exon3	Yes	Yes	JOAG	11	Caucasian, Asian	[12,27,32,33]
N450Y	Exon3		Yes	JOAG	20	China	Present study
T455K	Exon3		Yes	JOAG	26	China	[25]
Y471C	Exon3	Yes		NA	NA	China	[19,22]

The asterisk indicates uncertain pathogenicity and the double asterisk indicates the proband was in the end stage of glaucoma. NA refers date is unavailable.

**Figure 5:**
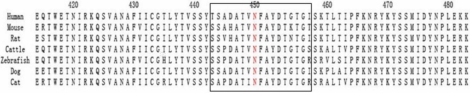
Sequence alignment portion of the olfactomedin-like domain spanning the novel missense mutation p.N450Y of human MYOC and a comparison with other species.

The Asn450 residue, located in the olfactomedin-like domain, is highly conserved in humans, rats, mice, cattle, dogs, and zabrafish (Figure 5). The results of GOR suggested that p.N450Y lead to a secondary structure change by replacing a coil structure with a β sheet around the Asn450 residue, which might interfere with the correct folding of the protein. In a large case control study, another mutation (p. N450D) was also detected at the Asn450 residue in a sporadic Germany patient [18]. This may imply that the Asn450 residue is very important for the activity of the olfactomedin-like domain.

Phenotype and genotype correlation has been well established in some *MYOC* mutations [11,12,27]. Patients carrying the P370L mutation usually developed glaucoma at a very early age, with high levels of IOP, which responds poorly to medical treatment [12,32,33]; while patients with the Q368X mutation were diagnosed with glaucoma at a later adult age and their maximum IOPs were around 30 mmHg, which could be well controlled by medical therapy [12,34,35]. One American family carrying the p.D380H MYOC mutation presented with an intermediate phenotype between juvenile and adult onset glaucoma [36]. In the current study, the onset age of glaucoma ranged from 20 to 31 years (mean 26 years). The mean highest IOP was 48.57 mmHg (range from 32 to 60 mmHg). One patient totally lost her sight before 35 years of age. Except for two patients newly diagnosed in 2009, the remaining five patients responded poorly to medical therapy and required filtration surgery for long-term IOP control. Five individuals diagnosed with ocular hypertension in 2004 carried the mutation p.N450Y and their mean age at diagnosis was 17.8 years. At the 5-year follow up, two of them presented glaucomatous optic disc change and were newly diagnosed with glaucoma. The phenotype and genotype correlation study on seven patients in this pedigree indicated that affected members carrying the mutation p.N450Y experienced more severe symptoms at an earlier age.

Incomplete penetrance has been observed in most families with *MYOC* mutations and the penetrances are age-dependent and mutation-specific [11,12,27]. The penetrance of pedigrees carrying p. P370L was 100% at age 30 years [12,32,33], while it was 0 for the pedigrees with Q368X [12,34,35]. In this pedigree, two clinically healthy individuals and three ocular hypertension patients were found harboring both mutation p.N450Y and the affected haplotype. The penetrance of this pedigree was 50% (6/12) at age 30 and almost 60% (7/12) at age 35 years. More than 80% (10/12) of the individuals carrying the p.N450Y mutation have developed glaucoma or ocular hypertension. Interestedly, one of the healthy members (II-12) was already 39 years old, which was ten years older than the average onset age of this family; this implied that other unidentified factors (genetic or environmental) might be associated with the JOAG of this pedigree. However, whole carriers should undergo ophthalmologic surveillance at regular intervals for the rest of their lives.

In summary, the report described a novel conserved tyrosine to asparagine substitution at exon 3 of *MYOC* associated with an early-onset and severe juvenile-onset open angle glaucoma pedigree. The results further expanded the mutation spectrum of *MYOC* and characterized the genotype-phenotype correlations of this pedigree. These results provide pre-symptomatic molecular diagnosis for the members of the pedigree and are useful for further genetic consultation with this family.
